# Protective and therapeutic effect of felodipine against bleomycin-induced pulmonary fibrosis in mice

**DOI:** 10.1038/s41598-017-03676-y

**Published:** 2017-06-13

**Authors:** Ken-ichiro Tanaka, Tomomi Niino, Tomoaki Ishihara, Ayaka Takafuji, Takahiro Takayama, Yuki Kanda, Toshifumi Sugizaki, Fumiya Tamura, Shota Kurotsu, Masahiro Kawahara, Tohru Mizushima

**Affiliations:** 1 Laboratory of Bio-Analytical Chemistry, Research Institute of Pharmaceutical Sciences, Faculty of Pharmacy, Musashino University, 1-1-20 Shinmachi, Nishitokyo-shi, Tokyo, Japan; 2 Division of Drug Discovery and Development, Faculty of Pharmacy, Keio University, Tokyo, Japan; 3 LTT Bio-Pharma Co., Ltd., Tokyo, Japan

## Abstract

Idiopathic pulmonary fibrosis (IPF) involves alveolar epithelial injury and abnormal collagen production caused by activated fibroblasts; transforming growth factor (TGF)-β1 is implicated in this activation. In this study, we screened for chemicals capable of inhibiting TGF-β1-induced collagen production in cultured fibroblasts from medicines already in clinical use. We selected felodipine based on its extent of collagen production inhibition, clinical safety profile, and other pharmacological activity. Felodipine is a dihydropyridine Ca^2+^ channel blocker that has been used clinically to treat patients with high blood pressure. Felodipine suppressed collagen production within LL29 cells in the presence of TGF-β1, but not in its absence. Intratracheal administration of felodipine prevented bleomycin-induced pulmonary fibrosis, alteration of lung mechanics and respiratory dysfunction. Felodipine also improved pulmonary fibrosis, as well as lung and respiratory function when administered after fibrosis development. Furthermore, administration of felodipine suppressed a bleomycin-induced increase in activated fibroblasts in the lung. We also found other dihydropyridine Ca^2+^ channel blockers (nifedipine and benidipine) inhibited collagen production *in vitro* and partially prevented bleomycin-induced pulmonary fibrosis, alteration of lung mechanics and respiratory dysfunction *in vivo*. We propose that these Ca^2+^ channel blockers may be therapeutically beneficial for IPF patients.

## Introduction

Idiopathic pulmonary fibrosis (IPF) is a progressive and devastating chronic lung condition with poor prognosis; mean length of survival from time of diagnosis is 2.8–4.2 years^1, 2^. Current agents for treatment of IPF, such as steroids and immunosuppressants, have not been found to improve prognosis^2–4^. Though two recently marketed drugs, pirfenidone and nintedanib, have been shown to significantly slow the decline of forced vital capacity (FVC) in clinical studies of IPF patients^2, 5, 6^, both of these drugs present severe adverse effects, especially within the gastrointestinal tract^5, 6^. Thus, safer drugs to treat IPF are required.

Upon injury to the lung, repair and remodeling processes, such as collagen synthesis, are induced to address this injury. However, in IPF patients, this process is excessively stimulated, which results in abnormal fibrosis characterised by collagen deposition^7, 8^. Transforming growth factor (TGF)-β1 reportedly plays an important role in the pathogenesis of IPF, especially with regard to abnormal fibrosis^8, 9^. Lung myofibroblasts, an intermediate cell type between fibroblast and smooth muscle cell, produce considerable amounts of extracellular matrix components like collagen, which may give rise to abnormal fibrosis^10^. TGF-β1 appears to stimulate both activation of fibroblasts into myofibroblasts and collagen synthesis^11, 12^. Thus, chemicals capable of suppressing TGF-β1-induced production of collagen represent good candidates to treat IPF patients. In fact, both pirfenidone and nintedanib have been reported to suppress collagen production in activated fibroblasts^13–15^.

The number of drugs reaching the marketplace each year is decreasing, mainly because unexpected adverse effects of potential drugs are revealed at later clinical trial stages. We have proposed a new strategy for drug discovery and development (drug re-positioning), which focuses on the use of existing medicines for alternative indications^16^. In this strategy, compounds with clinically beneficial pharmacological activity are screened from a library of medicines already in clinical use. The advantage of this strategy is a decreased risk for unexpected adverse effects in humans, as the safety aspects of these drugs have already been well-characterised^16^. Recently, we discovered new candidates for chronic obstructive pulmonary disease (COPD) and functional dyspepsia by this strategy^17–19^. Thus, this strategy is useful for discovering new candidates to treat human diseases.

In this study, we screened for small molecules capable of inhibiting TGF-β1-induced collagen production in cultured fibroblasts and identified felodipine from a library of medicines already in clinical use. Administration of felodipine, a dihydropyridine Ca^2+^ channel blocker previously used clinically to treat patients with high blood pressure^20^, showed both preventive and therapeutic effects against bleomycin-induced pulmonary fibrosis. We also found other dihydropyridine Ca^2+^ channel blockers (nifedipine and benidipine) inhibited collagen production *in vitro* and partially prevented bleomycin-induced pulmonary fibrosis *in vivo*. Notably, the concentrations of these drugs to prevent pulmonary fibrosis were higher than those required for Ca^2+^ channel inhibition. We propose that dihydropyridine Ca^2+^ channel blockers may be therapeutically beneficial for IPF patients.

## Results

### Effect of felodipine on collagen production in LL29 cells

From a library of medicines already in clinical use, we screened for compounds capable of inhibiting collagen production in the presence of TGF-β1 without affecting cell viability in NIH3T3, HFL1, WI-38 and IMR-90 cells. Among drugs that inhibited collagen production in all cell types, we selected felodipine based on its extent of collagen production inhibition, clinical safety profile, and other pharmacological activity (data not shown).

**Figure 1:**
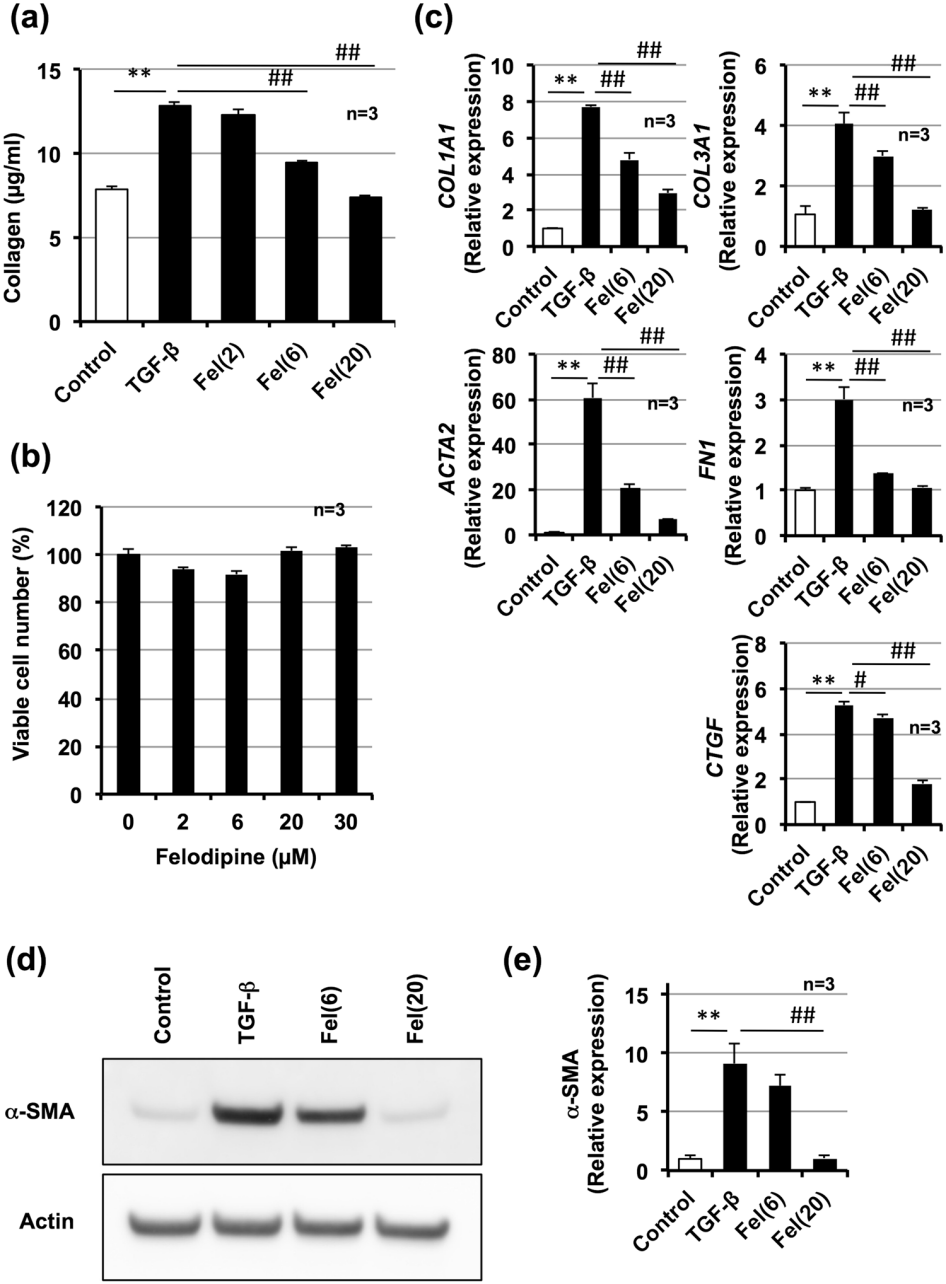
Effect of felodipine on TGF-β1-induced collagen production. LL29 cells were incubated with TGF-β1 (5 ng/ml) for 48 h (A, B) or 24 h (C, D) in the presence of indicated concentrations (µM) of felodipine (Fel). Collagen levels in culture medium were determined by Sircol assay (**a**). Viable cell number was determined by MTT method (**b**). Total RNA was extracted and subjected to real-time RT-PCR using a specific primer set for each gene. Values were normalised to *GAPDH* and expressed relative to control sample (**c**). Whole-cell extracts were analysed by immunoblotting with an antibody against *α*-SMA or actin (**d**). The *α*-SMA band intensity was determined using Image J software (**e**). Values represent mean ± S.E.M. ** or ^##^ *P* < *0*.*01* and ^#^ *P* < *0*.*05*. (* vs Control, # vs TGF-β).

As shown in Fig. 1a, treatment of LL29 cells with felodipine (more than 6 µM) in the presence of TGF-β1 decreased the amount of collagen within culture medium. Administration of felodipine (up to 30 µM) hardly affected the number of viable cells (Fig. 1b). We also examined the effect of felodipine on fibrosis-related genes expression. As shown in Fig. 1c, treatment of LL29 cells with TGF-β1 induced expression of *COL1A1*, *COL3A1*, *ACTA2* (α-SMA), *FN1* (fibronectin) and *connective tissue growth factor* (*CTGF*) mRNA; whereas, felodipine clearly suppressed the expression of these genes. As shown in Fig. 1d, treatment of LL29 cells with felodipine decreased protein expression of α-SMA in the presence of TGF-β1. Collectively, the results shown in Fig. 1 suggest felodipine suppresses synthesis of collagen at the transcriptional level. Further, as shown in Supplementary Fig. S1, treatment of HFL1 cells (healthy human lung fibroblasts) with felodipine also decreased TGF-β1-dependent expression of α-SMA protein. Compared to LL29 cells, TGF-β1-dependent increases in the expression of α-SMA were lower in HFL1 cells.

Phosphorylation of Smad3 is reportedly involved in the TGF-β signaling pathway^21, 22^. Thus, we examined the effect of felodipine on TGF-β signaling. As shown in Supplementary Fig. S2a, treatment of LL29 cells with TGF-β1 increased the expression of phospho-Smad3. In contrast, treatment of LL29 cells with felodipine clearly decreased TGF-β1-dependent increases in phospho-Smad3 (Supplementary Fig. S2b), suggesting that felodipine suppresses TGF-β signaling via suppressing phosphorylation of Smad3. Moreover, as shown in Supplementary Fig. S3, treatment of HFL1 cells with felodipine also decreased TGF-β1-dependent increases in phospho-Smad3. Compared to LL29 cells, the TGF-β1-dependent increases in the expression of phospho-Smad3 were lower in HFL1 cells.

### Preventive effect of felodipine on bleomycin-induced pulmonary fibrosis

**Figure 2:**
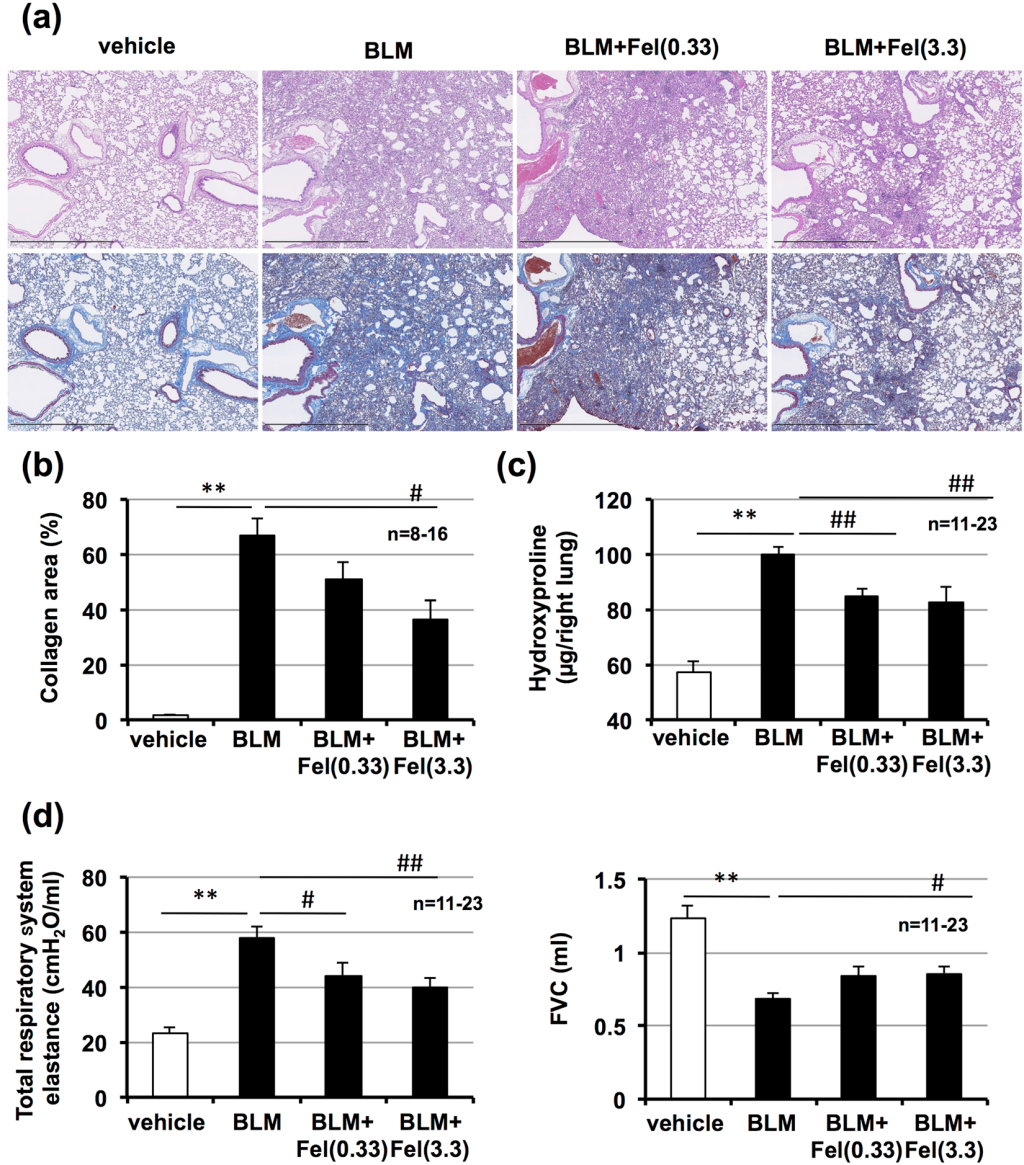
Effect of felodipine on bleomycin-induced pulmonary fibrosis, alteration of lung mechanics, and respiratory dysfunction. Mice were intratracheally administered with bleomycin (BLM, 2 mg/kg) or vehicle once only on day 0. Mice were intratracheally administered indicated doses (mg/kg) of felodipine (Fel) once daily for 14 days (from day 0–13). Sections of pulmonary tissue were prepared on day 14 (24 h after final felodipine administration) and subjected to histopathological examination including H&E staining (upper images) and Masson’s trichrome staining (lower images). Scale bar = 1.0 mm (**a**). Percentage collagen-positive area was determined based on images of Masson’s trichrome staining (**b**). Pulmonary hydroxyproline level was determined on day 14 (**c**). Total respiratory system elastance and FVC were measured on day 14 (**d**). Values represent mean ± S.E.M. ** or ^##^ *P* < *0*.*01* and ^#^ *P* < *0*.*05*. (* vs vehicle, # vs BLM).

Pulmonary fibrosis was induced in mice with intratracheal administration of bleomycin. Histopathological analysis of pulmonary tissue by H&E and Masson’s trichrome staining revealed bleomycin-induced severe pulmonary damage (thickened and edematous alveolar walls and interstitium) and collagen deposition, and that these lesions were suppressed by intratracheal administration of felodipine (Fig. 2a). Felodipine-dependent suppression of bleomycin-induced pulmonary fibrosis was also confirmed by determination of collagen area (based on images of Masson’s trichrome staining, as shown in Fig. 2b) and pulmonary hydroxyproline content (Fig. 2c), both indicators of fibrosis^23^.

Changes in lung mechanics associated with pulmonary fibrosis are characterised by an increase in elastance^24^. Total respiratory system elastance (including bronchi, bronchioles, and alveoli) and tissue elastance (alveoli only) increased following bleomycin treatment. These effects were partially relieved by administration of felodipine (Fig. 2d). Using a computer-controlled ventilator and negative pressure reservoir, we found FVC decreased in bleomycin-treated mice and felodipine treatment suppressed this effect (Fig. 2d). These results show intratracheal administration of felodipine protected against bleomycin-induced pulmonary fibrosis and resulting alterations in lung mechanics and respiratory function.

### Therapeutic effect of felodipine on bleomycin-induced pulmonary fibrosis

**Figure 3:**
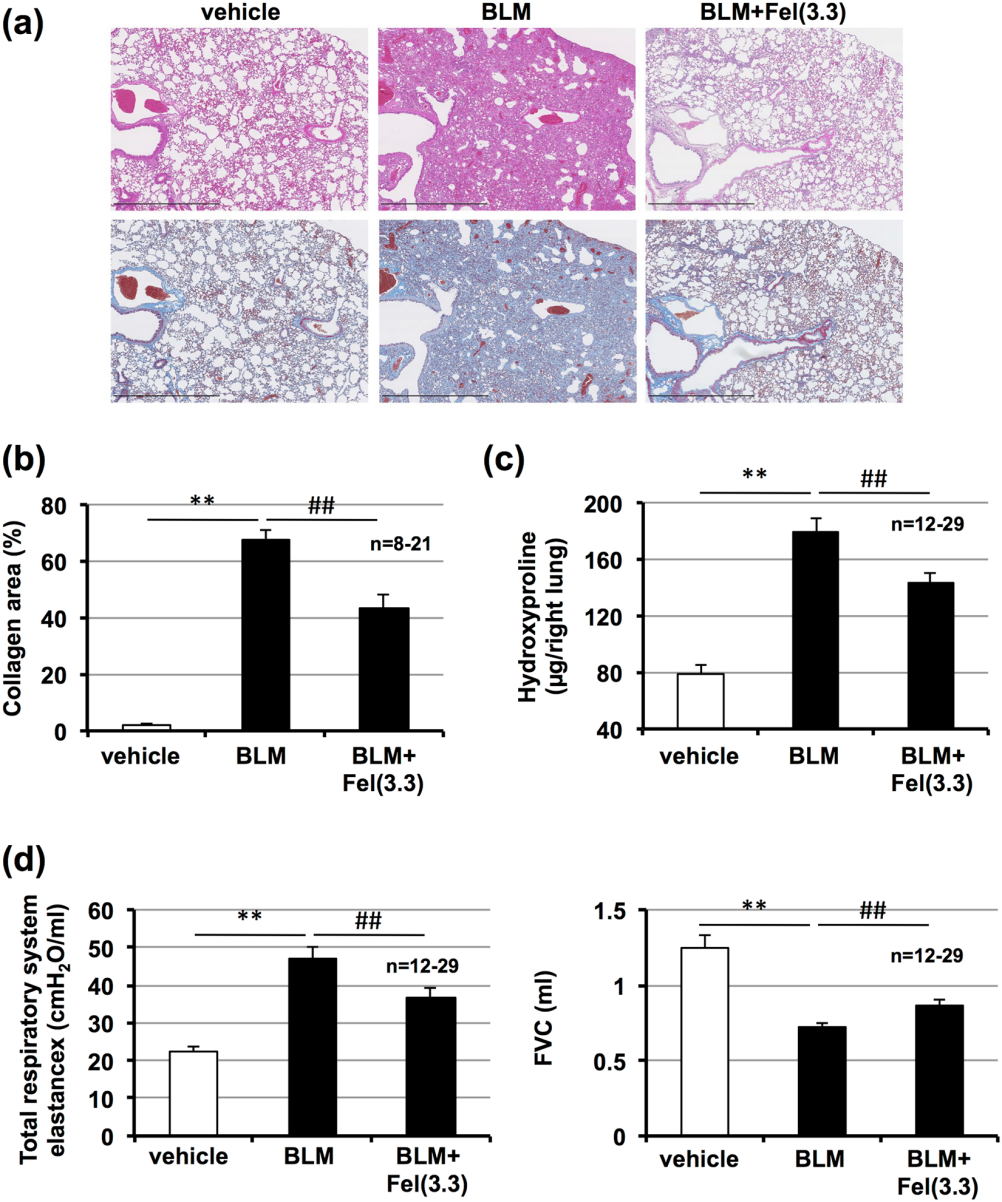
Effect of felodipine on pre-developed pulmonary fibrosis. Mice were intratracheally administered with bleomycin (BLM, 1 mg/kg) or vehicle once only on day 0. Mice were intratracheally administered indicated doses (mg/kg) of felodipine (Fel) once daily for 10 days (from day 10–19). Sections of pulmonary tissue were prepared on day 21 (48 h after final felodipine administration) and subjected to histopathological examination including H&E staining (upper images) and Masson’s trichrome staining (lower images). Scale bar = 1.0 mm (**a**). Percentage of collagen-positive area was determined based on images of Masson’s trichrome staining (**b**). Pulmonary hydroxyproline level was determined on day 21 (**c**). Total respiratory system elastance and FVC were measured on day 21 (**d**). Values represent mean ± S.E.M. ** or ^##^ *P* < *0*.*01*; ^#^ *P* < *0*.*05*. (* vs vehicle, # vs BLM).

We next tested the efficacy of a felodipine treatment protocol after the development of fibrosis. Drug treatment commenced 10 days after administration of bleomycin and pulmonary fibrosis, and then lung mechanics and FVC were assessed on day 20. We first confirmed the presence of pulmonary fibrosis on day 10 (data not shown). As shown in Fig. 3a–c, administration of felodipine decreased the extent of pulmonary damage and fibrosis. We also found that administration of felodipine significantly decreased lung elastance and increased FVC on day 20 (Fig. 3d). These data suggest felodipine could be an effective agent for treatment of pre-existing pulmonary fibrosis.

**Figure 4:**
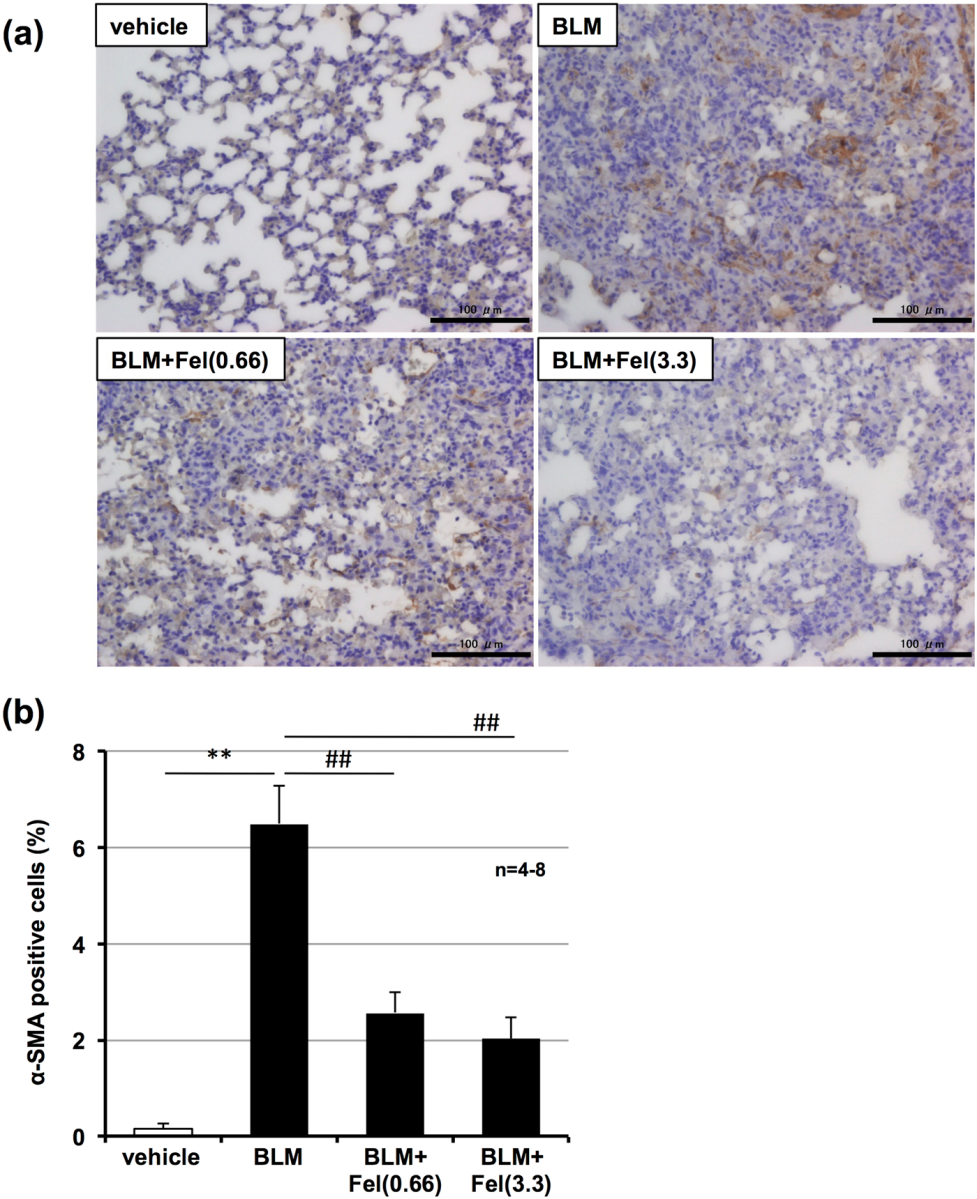
Effect of felodipine on bleomycin-induced increase in myofibroblasts. Mice were intratracheally administered with bleomycin (BLM, 1 mg/kg) or vehicle once only on day 0. Mice were intratracheally administered indicated doses (mg/kg) of felodipine (Fel) once daily for 3 days (from day 10–12). Sections of pulmonary tissue were prepared on day 13 (24 h after final felodipine administration) and subjected to immunohistochemical analysis with an antibody against α-SMA (**a**,**b**). Scale bar = 100 μm. Percentage of area stained with antibody was determined using ImageJ software (**b**). Values represent mean ± S.E.M. ** or ^##^ *P* < *0*.*01*. (* vs vehicle, # vs BLM).

As described in the introduction, myofibroblasts play an important role in pulmonary fibrosis in IPF patients and bleomycin-induced pulmonary fibrosis in animals^10, 25^. As such, we used immunostaining to monitor levels of α-SMA, a marker for myofibroblasts^10^. As shown in Fig. 4a,b, administration of bleomycin increased the number of α-SMA-positive cells in the lung (mainly around main bronchus) and felodipine suppressed this increase. These results suggest felodipine suppresses the differentiation of fibroblasts to myofibroblasts. Further, we also monitored levels of phospho-Smad3 *in vivo*. As shown in Supplementary Fig. S4a,b, administration of bleomycin increased the number of phospho-Smad3-positive cells in the lung and felodipine suppressed this increase, suggesting that felodipine also suppresses the phosphorylation of Smad3 *in vivo*.

### Effect of other Ca^2+^ channel blockers on collagen production *in vitro* and bleomycin-induced pulmonary fibrosis *in vivo*

**Figure 5:**
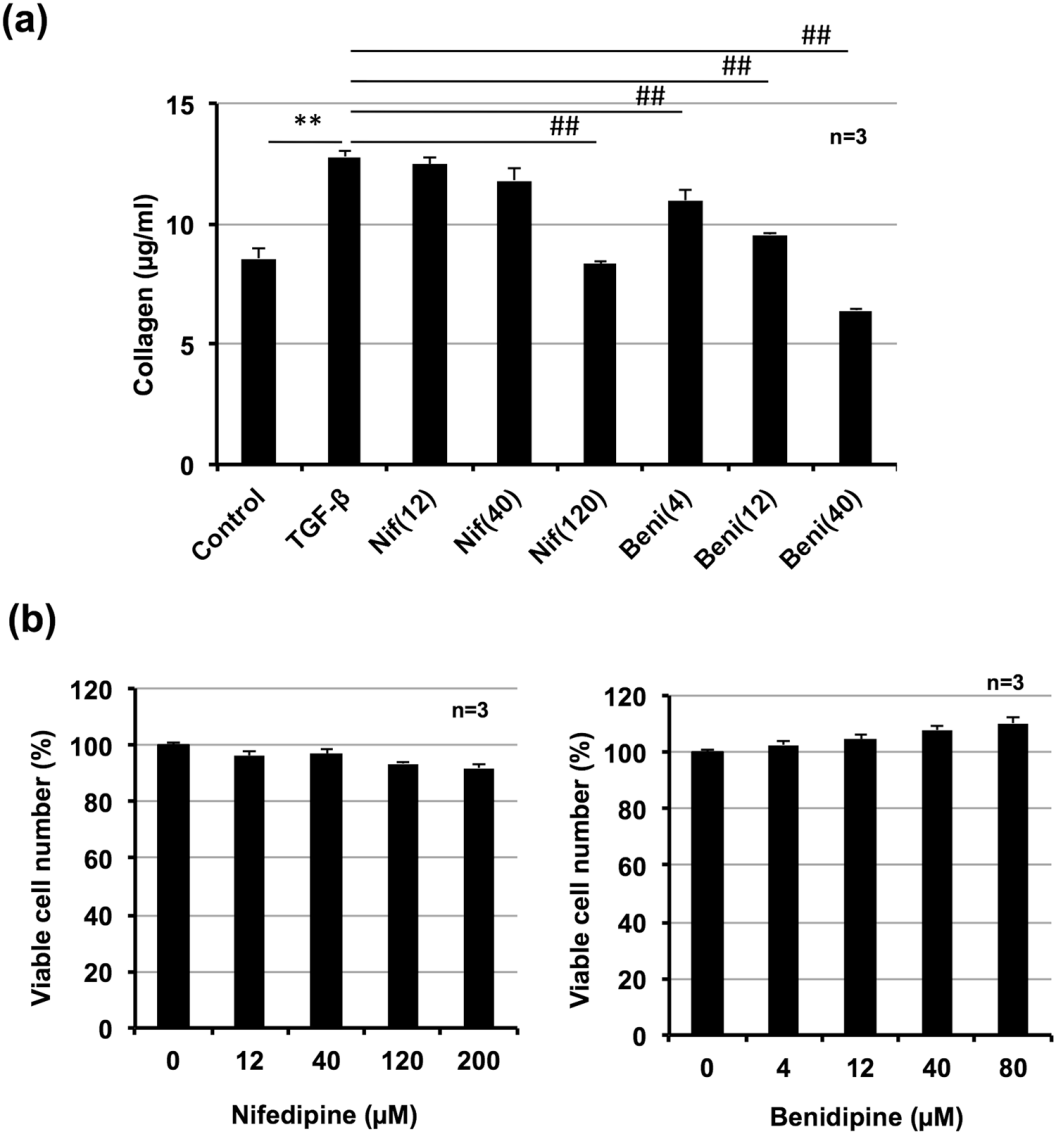
Effect of other calcium channel blockers on TGF-β1-induced collagen production. LL29 cells were incubated with TGF-β1 (5 ng/ml) for 48 h (**a**) or 24 h (**b**) in the presence of indicated concentrations (µM) of nifedipine (Nif) or benidipine (Beni). Level of collagen in culture medium was determined by Sircol assay (**a**). Viable cell number was determined by MTT method (**b**). Values represent mean ± S.E.M. ** or ^##^ *P* < *0*.*01*. (* vs Control, # vs TGF-β).

As we were performing experiments for this study, Mukherjee *et al*. reported that nifedipine and other Ca^2+^ channel blockers suppressed bleomycin-induced pulmonary fibrosis through modulation of Ca^2+^ oscillations evoked by TGF-β1^26^. Thus, we tested the effect of other Ca^2+^ channel blockers (nifedipine and benidipine) in our *in vitro* and *in vivo* systems. As shown in Fig. 5a, treatment with either nifedipine (120 µM) or benidipine (more than 4 µM) decreased the amount of collagen in LL29 cell culture medium with TGF-β1. We confirmed that neither nifedipine nor benidipine affected cell viability at these doses (Fig. 5b).

**Figure 6:**
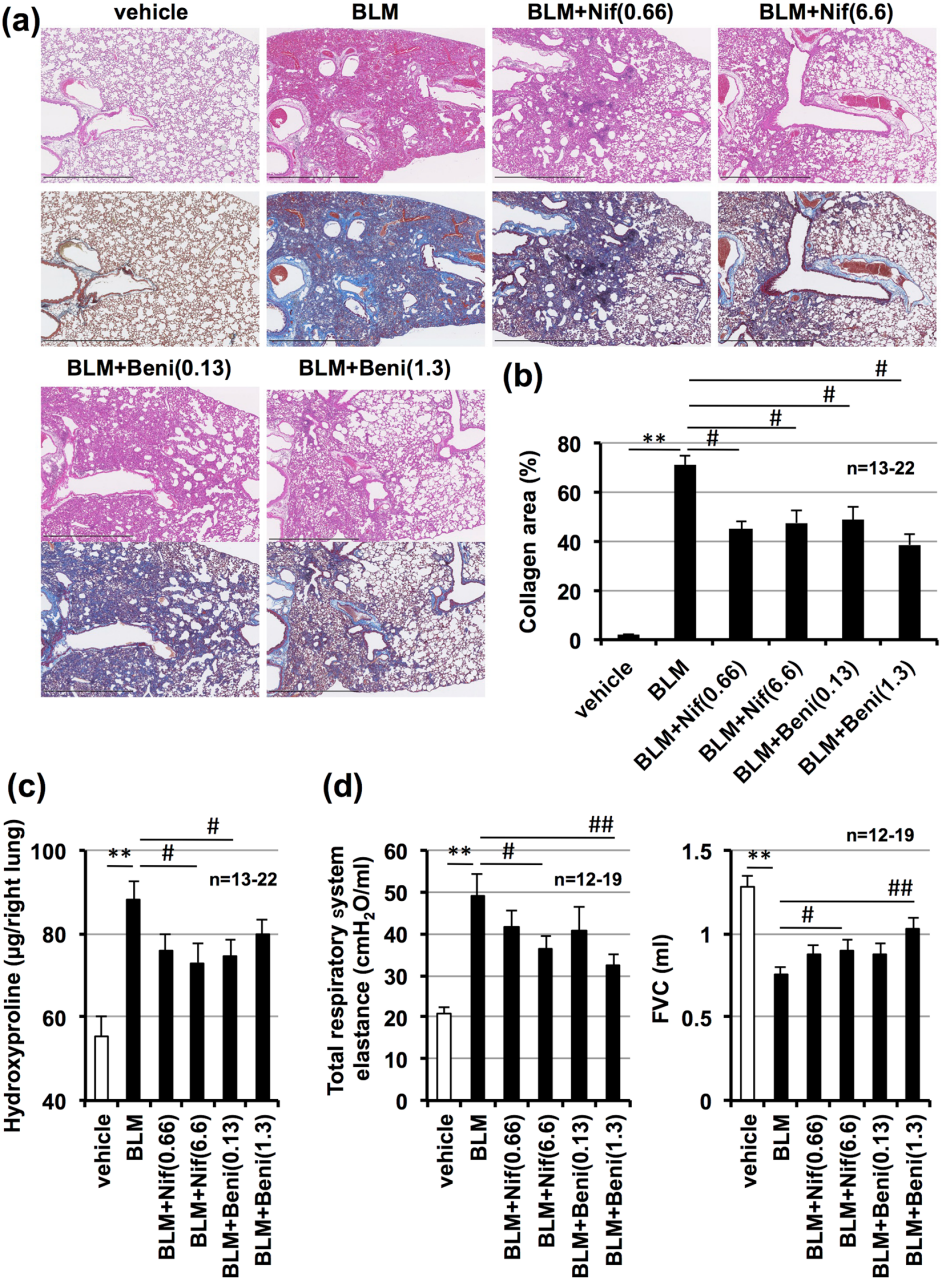
Effect of other calcium blockers on bleomycin-induced pulmonary fibrosis, alteration of lung mechanics, and respiratory dysfunction. Mice were intratracheally administered with bleomycin (BLM, 2 mg/kg) or vehicle once only on day 0. Mice were intratracheally administered indicated doses (mg/kg) of nifedipine (Nif) or benidipine (Beni) once daily for 14 days (from day 0–13). Sections of pulmonary tissue were prepared on day 14 (24 h after final calcium blockers administration) and subjected to histopathological examination. H&E staining (upper images) and Masson’s trichrome staining (lower images); scale bar = 1.0 mm (**a**). Percentage of collagen-positive area was determined based on images of Masson’s trichrome staining (**b**). Pulmonary hydroxyproline level was determined on day 14 (**c**). Total respiratory system elastance and FVC were measured on day 14 (**d**). Values represent mean ± S.E.M. ** or ^##^ *P* < *0*.*01* and ^#^ *P* < *0*.*05*. (* vs vehicle, # vs BLM).

We then tested the efficacy of nifedipine or benidipine on bleomycin-induced pulmonary fibrosis. As shown in Fig. 6a–c, administration of nifedipine or benidipine decreased the extent of bleomycin-induced pulmonary damage and fibrosis. We also found administration of nifedipine or benidipine significantly decreased lung elastance and increased FVC, with the exception of tissue elastance associated with nifedipine (Fig. 6d). Considering results shown in Figs 5 and 6, we propose dihydropyridine Ca^2+^ channel blockers may be therapeutically beneficial for IPF patients.

## Discussion

In this study, we identified felodipine as a chemical inhibitor of TGF-β1-induced collagen production *in vitro* from medicines already in clinical use. Felodipine suppressed collagen production in LL29 cells (IPF patient lung fibroblasts) in the presence of TGF-β1. Intratracheal administration of felodipine provided both preventive and therapeutic effects against bleomycin-induced pulmonary fibrosis, including restoration of lung mechanics, improvement of respiratory dysfunction, and decrease of activated fibroblasts in the lung. We also found other Ca^2+^ channel blockers (nifedipine and benidipine) inhibited collagen production *in vitro* and prevented bleomycin-induced pulmonary fibrosis *in vivo*. Collectively, we propose felodipine and other dihydropyridine Ca^2+^ channel blockers may be therapeutically beneficial for IPF patients.

As shown in Supplementary Figs S2 and S4, treatment of LL29 cells with felodipine inhibited TGF-β1- or bleomycin-dependent increases in phospho-Smad3, suggesting that felodipine suppresses TGF-β1-dependent myofibroblast differentiation and/or collagen production through decreased phosphorylation of Smad3. However, we have not fully examined the molecular mechanisms of felodipine in this study. Recently, Mukherjee *et al*. reported that TGF-β1 stimulates collagen production not only through Smad3 signaling pathways, but also through propagation of periodic oscillations in Ca^2+^; further, Ca^2+^ channel blockers are capable of suppressing periodic Ca^2+^ oscillations in human fibroblasts^26, 27^. Considering these results, molecular mechanisms underlying felodipine-dependent suppression of myofibroblast differentiation or inhibition of collagen production may be partially mediated by propagation of periodic oscillations in Ca^2+^. On the other hand, the concentrations of Ca^2+^ channel blockers necessary to inhibit collagen production (e.g. 6 or 20 µM felodipine) were higher than those required for L-type Ca^2+^ channel inhibition (e.g. 0.15 nM felodipine^28^). In addition, it is unclear if functional voltage-gated L-type Ca^2+^ channels exist within target cells producing the observed protective effect (most likely myofibroblasts). Therefore, the protective effects of these Ca^2+^ blockers may occur by other low affinity molecular targets [e.g. micromolar concentrations of Ca^2+^ blockers have been shown to activate the transient receptor potential melastatin-3 (TRPM3) channel^29^].

To develop treatments for IPF, it is important to examine both preventive and therapeutic effects of felodipine on bleomycin-induced pulmonary fibrosis. Further, two recently developed IPF drugs, pirfenidone and nintedanib, have been reported to treat bleomycin-induced pre-developed pulmonary fibrosis^15, 30^. In this study, we found that felodipine possesses both preventive and therapeutic effects against bleomycin-induced pulmonary fibrosis (Figs 2 and 3). To our knowledge, this is the first time that the therapeutic effect of felodipine against pulmonary fibrosis has been confirmed. Moreover, as the diagnosis of IPF in human patients is confirmed by a decrease in FVC^2^, it is important to examine the effect of candidate drugs on respiratory function related to FVC in animal models of IPF. To this end, we showed felodipine clearly improved bleomycin-induced decreases in FVC. Thus, felodipine may be therapeutically beneficial for IPF patients. Furthermore, we propose that repeated inhalation of felodipine or other dihydropyridine Ca^2+^ channel blockers may also be an attractive candidate for preventing this severe side effect of bleomycin therapy for cancer patients. Notably, lower parts of the lung seem to be improved by bleomycin-induced pulmonary fibrosis even though felodipine or other Ca^2+^ channel blockers (Fig. 6)) were administered intratracheally. However, as it is reportedly difficult to uniformly produce bleomycin-induced pulmonary fibrosis^31^, we considered the heterogeneous distribution of anti-fibrotic effects elicited by Ca^2+^ channel blockers to be caused by the heterogeneity of bleomycin-induced pulmonary fibrosis.

Some investigations reported that Ca^2+^ channel blockers have anti-inflammatory effects in various models. For example, verapamil suppressed TNF-α-induced expression of inflammatory cytokines *in vitro* and reduced levels of inflammatory cytokines in mice arthritis models^32^. Moreover, felodipine attenuated vascular inflammation in a rat model of metabolic syndrome^33^. Thus, we examined whether felodipine exerts anti-inflammatory effects in our experimental conditions. As shown in Supplementary Fig. S5a, the total number of leucocytes, and the numbers of macrophages, neutrophils and lymphocytes were increased by the bleomycin treatment, and these effects (except for lymphocytes) were suppressed by simultaneous felodipine administration (from day 0 to 2, “Preventive study”). These results suggested that felodipine prevented bleomycin-induced pulmonary fibrosis by decreasing the bleomycin-induced pulmonary inflammation. In contrast, felodipine administration (from day 10 to 12, “Therapeutic study”) could not suppress bleomycin-induced pulmonary inflammation (Supplementary Fig. S5b). These results support our view that felodipine treated bleomycin induced pulmonary fibrosis by suppressing the TGF-β1-dependent collagen production.

Activation of the TGF-β signaling pathway is reportedly involved in other pulmonary diseases, such as severe asthma, COPD, and pulmonary arterial hypertension^34–36^. Therefore, the inhibitory effect of TGF-β1-induced collagen production by Ca^2+^ channel blockers may also be important for treatment of other pulmonary diseases. Indeed, Girodet *et al*. showed that treatment for 12 months with a Ca^2+^ channel blocker, gallopamil, reduced bronchial smooth muscle remodeling and prevented the occurrence of asthma exacerbations in patients with severe asthma^37^. Considering these reports, the results of the current study would be useful for establishing treatment strategies for other pulmonary diseases. Finally, based on our findings in this study, we propose felodipine and other dihydropyridine Ca^2+^ channel blockers may be therapeutically beneficial for IPF patients, as safety for use in humans has already been clinically confirmed.

## Materials and Methods

### Chemicals, reagents, and animals

Chloramine T, 3-(4,5-dimethylthiazol-2-yl)-2,5-diphenyltetrazolium bromide (MTT), 4-(dimethylamino)-benzaldehyde (DMBA), potassium dichromate, phosphotungstic acid, phosphomolybdic acid, Orange G and acid fuchsin were obtained from Sigma-Aldrich (St. Louis, MO). Fetal bovine serum (FBS) was purchased from BioWest (Nuaille, France); whereas, Ham’s F-12K (Kaighn’s) Medium and Minimum Essential Medium (MEM) were from Thermo Fisher Scientific (Waltham, MA). Dulbecco’s Modified Eagle Medium (DMEM) was from Nissui Pharmaceutical Co., Ltd. (Tokyo, Japan). Antibodies against actin, or α-smooth muscle actin (α-SMA) were purchased from Santa Cruz Biotechnology (Santa Cruz, CA), or Abcam (Cambridge, UK). Antibodies against phospho-Smad3 was purchased from Cell Signaling Technology (Tokyo, Japan). Felodipine was from Tokyo Chemical Industry (Tokyo, Japan) and bleomycin was from Nippon Kayaku (Tokyo, Japan). Sircol™ Collagen Assay Kit was from Biocolor (Antrim, UK) and chloral hydrate was from Nacalai Tesque (Kyoto, Japan). Nifedipine, benidipine, isoflurane, L-hydroxyproline, sodium acetate, trichloroacetic acid (TCA), perchloric acid, azophloxin, aniline blue, and formalin neutral buffer solution were obtained from Wako Pure Chemicals (Tokyo, Japan). Mayer’s hematoxylin, 1% eosin alcohol solution, mounting medium (malinol) and Weigert’s iron hematoxylin were from Muto Pure Chemicals (Tokyo, Japan). Xylidine ponceau was from Chroma/Waldeck (Münster, Germany). RNeasy® kit was obtained from Qiagen (Hilden, Germany), PrimeScript® 1^st^ strand cDNA Synthesis Kit was from Takara Bio (Ohtsu, Japan), and SsoFast^TM^ EvaGreen Supermix was from Bio-Rad (Hercules, CA). All cells were purchased from the American Type Culture Collection (Manassas, VA). ICR mice (6–7 weeks old, male) were purchased from Charles River (Yokohama, Japan). Experiments and procedures described herein were approved by the Animal Care Committee of Keio University or Musashino University and carried out in accordance with the Guide for the Care and Use of Laboratory Animals as adopted and promulgated by the National Institutes of Health.

### Treatment of mice with bleomycin and Ca^2+^ channel blockers

Mice maintained under anaesthesia with isoflurane were intratracheally administered bleomycin (1 mg/kg or 2 mg/kg) in 0.9% NaCl (2 ml/kg) and/or Ca^2+^ channel blockers (various doses) in 0.9% NaCl (1 ml/kg) via a P200 micropipette. For example, a mouse (body weight = 30 g) was intratracheally administered 30 µL of felodipine (0.33 or 3.3 mg/ml). The first administration of Ca^2+^ channel blockers was performed 1 h after bleomycin administration (except for experiments examining therapeutic effect, in which first administration of Ca^2+^ channel blockers was performed 10 days after bleomycin administration).

In control experiments, we examined the effect of administering felodipine (3.3 mg/kg) alone for 14 days, and found that it did not affect lung histology, lung mechanics, respiratory function, or the amount of hydroxyproline in the lung (Supplementary Fig. S6).

### Cell culture

LL29 cells (IPF patient lung fibroblasts) and HFL1 cells (healthy human lung fibroblasts) were cultured in Ham’s F-12K (Kaighn’s) Medium supplemented with 15% FBS. NIH3T3 cells (embryonic mouse fibroblasts) were cultured in DMEM supplemented with 10% FBS. WI-38 and IMR-90 cells (both healthy human lung fibroblasts) were cultured in MEM supplemented with 10% FBS. All cells were cultured in a humidified atmosphere of 95% air with 5% CO_2_ at 37 °C. Viable cell number was monitored by MTT method^38^. Amounts of collagen in culture media were measured with a Sircol™ Collagen assay kit according to the manufacturer’s protocol. All experiments in which TGF-β1 or Ca^2+^ channel blockers were added were performed using culture medium supplemented without FBS.

### Measurement of lung mechanics and FVC

Lung mechanics and FVC were measured with a computer-controlled small-animal ventilator (flexiVent^TM^; SCIREQ, Montreal, Canada), as previously described^23^. For lung mechanics, a tracheotomy was performed by anaesthetizing mice with chloral hydrate (500 mg/kg) and inserting an 8-mm long section of metallic tube (outer and inner diameters of 1.27 mm and 0.84 mm, respectively) into the trachea. Mice were mechanically ventilated at a rate of 150 breaths/min, using a tidal volume of 8.7 ml/kg and a positive end-expiratory pressure of 2–3 cmH_2_O. Total respiratory system elastance and tissue elastance were measured by snapshot and forced oscillation techniques, respectively. Determination of FVC was also performed using anaesthetized, tracheotomised and ventilated mice, as described above. Lungs were inflated to 30 cmH_2_O over one second and held at this pressure. After 0.2 s, the pinch valve (connected to the ventilator) was closed. After 0.3 s, the shutter valve (connected to the negative pressure reservoir) was opened, exposing the lung to negative pressure, which was held for 1.5 s to ensure complete expiration. All lung mechanic and FVC data were analysed using flexiVent software version 5.3 (SCIREQ).

### Hydroxyproline determination

Hydroxyproline content was determined as previously described^39^. Briefly, lungs were removed and homogenised in 0.5 ml of 5% TCA. After centrifugation, pellets were hydrolysed in 0.5 ml of 10N HCl for 16 h at 110 °C. After addition of 0.5 ml of 1.4% w/v chloramine T solution, each sample was incubated for 20 min at room temperature. Next, 0.5 ml of Ehrlich’s reagent (1M DMBA, 70% v/v isopropanol and 30% v/v perchloric acid) was added to samples, which were then incubated at 65 °C for 10 min. Absorbance was measured at 550 nm and amount of hydroxyproline was determined.

### Preparation of BALF

BALF was collected by cannulating the trachea and lavaging the lung twice with 1 ml of sterile 0.9% NaCl containing 50 units/ml heparin. Approximately 1.8 ml of BALF was routinely recovered from each mouse and the total cell number was counted using a hemocytometer. After centrifugation with a Cytospin®4 (Thermo Fisher Scientific, Waltham, MA), cells were stained with Diff-Quik reagents (Sysmex, Kobe, Japan) and the ratios of macrophages, neutrophils and lymphocytes to total cell number was determined.

### Real-time RT-PCR analysis

Total RNA was extracted from LL29 cells using an RNeasy kit according to the manufacturer’s protocol. Samples were reverse-transcribed using previously described kit and synthesised cDNA was used in real-time RT-PCR experiments with SsoFast EvaGreen Supermix and analysed with Bio-Rad’s CFX96™ Real-time system and CFX Manager™ software (Hercules, CA). Specificity was confirmed by electrophoretic analysis of reaction products and by the inclusion of template- or reverse transcriptase-free controls. To normalise the amount of total RNA present in each reaction, glyceraldehyde-3-phosphate dehydrogenase (GAPDH) cDNA was used as an internal standard. Primers were designed using Primer-BLAST websites. Primers sequences will be provided upon request.

### Immunoblotting analysis

Whole-cell extracts were prepared. The protein concentration of samples was determined using the Bradford method. Samples were applied to polyacrylamide SDS gels, subjected to electrophoresis, and the resultant proteins immunoblotted with their respective antibodies. Band intensities were quantitated by using ImageJ software (version 1.39 u).

### Histological and immunohistochemical analyses

Lung tissue samples were fixed in 10% formalin neutral buffer solution for 24 h. After fixing, samples were embedded in paraffin before being cut into 4-μm-thick sections. For histological examination, sections were stained first with Mayer’s hematoxylin and then with 1% eosin alcohol solution (H&E staining). For collagen staining (Masson’s trichrome staining), sections were treated sequentially with 5% w/v potassium dichromate and 5% w/v TCA; Weigert’s iron hematoxylin; 1.25% w/v phosphotungstic acid and 1.25% w/v phosphomolybdic acid; 0.75% w/v Orange G solution; 0.12% w/v xylidine ponceau, 0.04% w/v acid fuchsin and 0.02% w/v azophloxin; 2.5% w/v phosphotungstic acid; and finally, aniline blue solution. Samples were mounted with malinol and scanned using a NanoZoomer-XR digital slide scanner (Hamamatsu Photonics, Shizuoka, Japan) or visualizsed with a microscope and digital camera (Olympus DP71; Tokyo, Japan). ImageJ software (http://imagej.nih.gov/ij/) was used to measure collagen-positive areas of left lungs from all mice [n = 8–16 (Fig. 2), n = 8–21 (Fig. 3), n = 13–22 (Fig. 6)]. An investigator blinded to the study protocol conducted this quantification.

For immunohistochemical analysis of α-SMA or phospho-Smad3, sections were incubated with Tris-EDTA buffer (pH9.0) for antigen retrieval and incubated with DAKO® Peroxidase Blocking Reagent for removal of endogenous peroxidase. Sections were blocked with 3% goat serum for 10 min, incubated for 12 h with α-SMA antibody (1:50 dilution) or phospho-Smad3 antibody (1:50 dilution) (in DAKO® Antibody Diluent, and then incubated with peroxidase-labelled polymer conjugated to goat anti-rabbit immunoglobulins for 1 h. Then, 3, 3-diaminobenzidine was applied to the sections and the sections were finally incubated with Mayer’s hematoxylin. Samples were mounted with malinol and visualised with a microscope and digital camera (Olympus DP71; Tokyo, Japan). ImageJ software was used to measure α-SMA or phospho-Smad3-positive cells, as appropriate.

### Statistical analysis

All values are expressed as mean ± standard error of the mean (S.E.M.). One-way ANOVA followed by Dunnett’s test, or Student’s *t*-test for unpaired results, was used to evaluate differences between three or more groups, or between two groups, respectively. SPSS22 software (IBM, Armonk, NY) was used for all statistical analyses. Differences were considered to be significant for values of *P* < 0.05.

## Electronic supplementary material

Supple
