# Supplementary material for: Protective and therapeutic effect of felodipine against bleomycin-induced pulmonary fibrosis in mice
Source: Sci Rep. 2017 Jun 13;7:3439. doi: 10.1038/s41598-017-03676-y (PMC5469778; doi:10.1038/s41598-017-03676-y)
Supplement: Supplementary file 1 — Supple [file 41598_2017_3676_MOESM1_ESM.pdf]

Protective and therapeutic effect of felodipine against bleomycin-induced pulmonary fibrosis in mice

KEN-ICHIRO TANAKA[1,2]*, TOMOMI NIINO[2]*, TOMOAKI ISHIHARA[2]*, AYAKA TAKAFUJI[1], TAKAHIRO TAKAYAMA[1], YUKI KANDA[1], TOSHIFUMI SUGIZAKI[2], FUMIYA TAMURA[2], SHOTA KUROTSU[2], MASAHIRO KAWAHARA[1] AND TOHRU MIZUSHIMA[3]

[1]Laboratory of Bio-Analytical Chemistry, Research Institute of Pharmaceutical Sciences, Musashino University, 1-1-20 Shinmachi, Nishitokyo-shi, Tokyo, Japan. [2]Division of Drug Discovery and Development, Faculty of Pharmacy, Keio University, Tokyo, Japan, and [3]LTT Bio-Pharma Co., Ltd., Tokyo, Japan

Correspondence: Dr. Ken-ichiro Tanaka, Laboratory of Bio-Analytical Chemistry, Research Institute of Pharmaceutical Sciences, Musashino University, 1-1-20 Shinmachi, Nishitokyo-shi, Tokyo 202-8585, Japan. Tel/Fax: +81-42-468-9335; E-mail: k-tana@musashino-u.ac.jp

*KT, TN and TI contributed equally to this study.

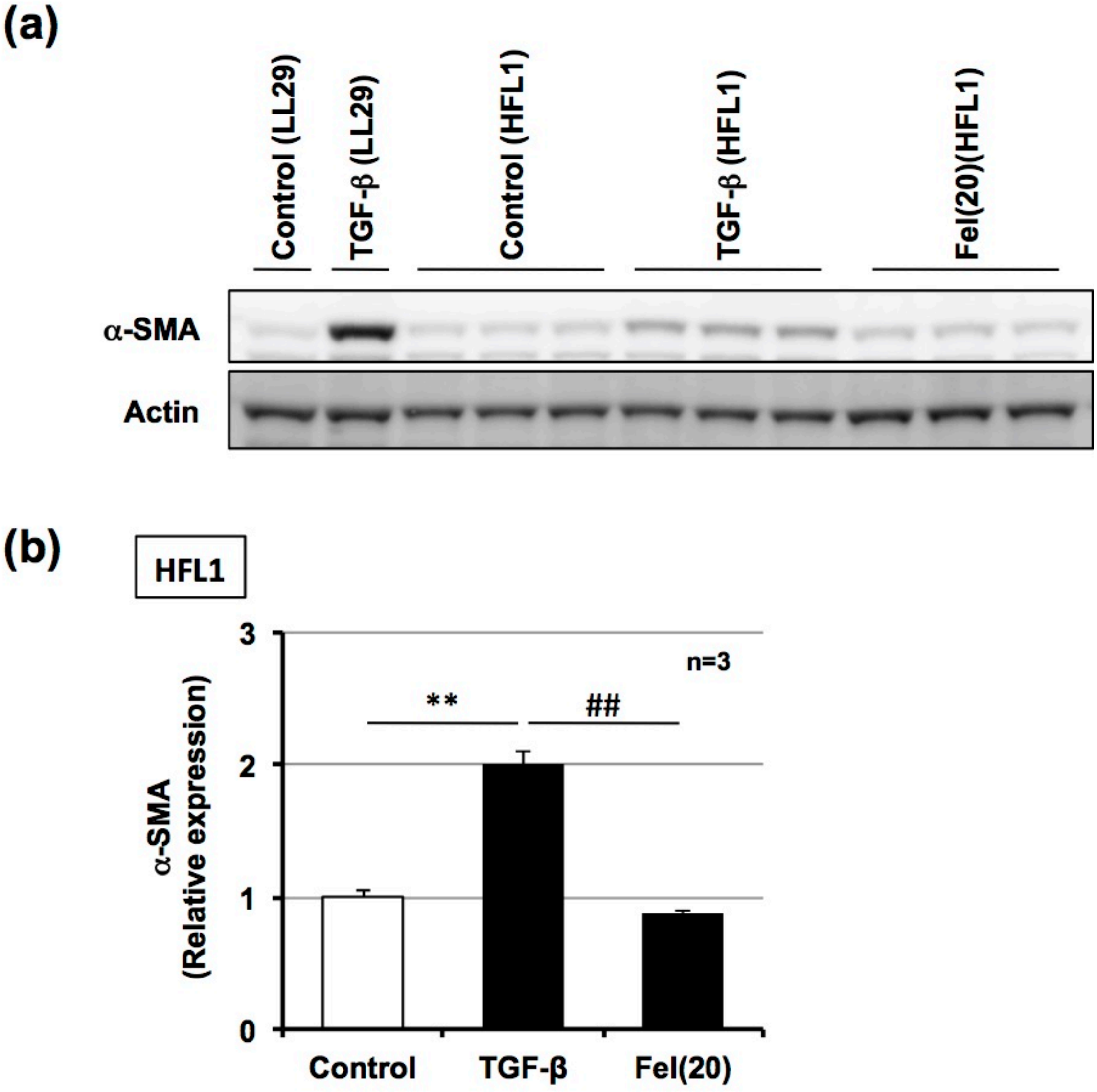

Supplementary Fig. S1. Effect of felodipine on TGF-β1-induced expression of α-SMA in HFL1 cells.

HFL1 cells or LL29 cells were incubated with TGF-β1 (5 ng/ml) for 24 h in the presence of felodipine (Fel, 20 μM). Whole-cell extracts were analysed by immunoblotting with an antibody against α-SMA or actin (a). The α-SMA band intensity was determined using Image J software (b). Values represent mean ± S.E.M. ** or $^{##}$*P < 0.01*. (* vs Control, # vs TGF-β).

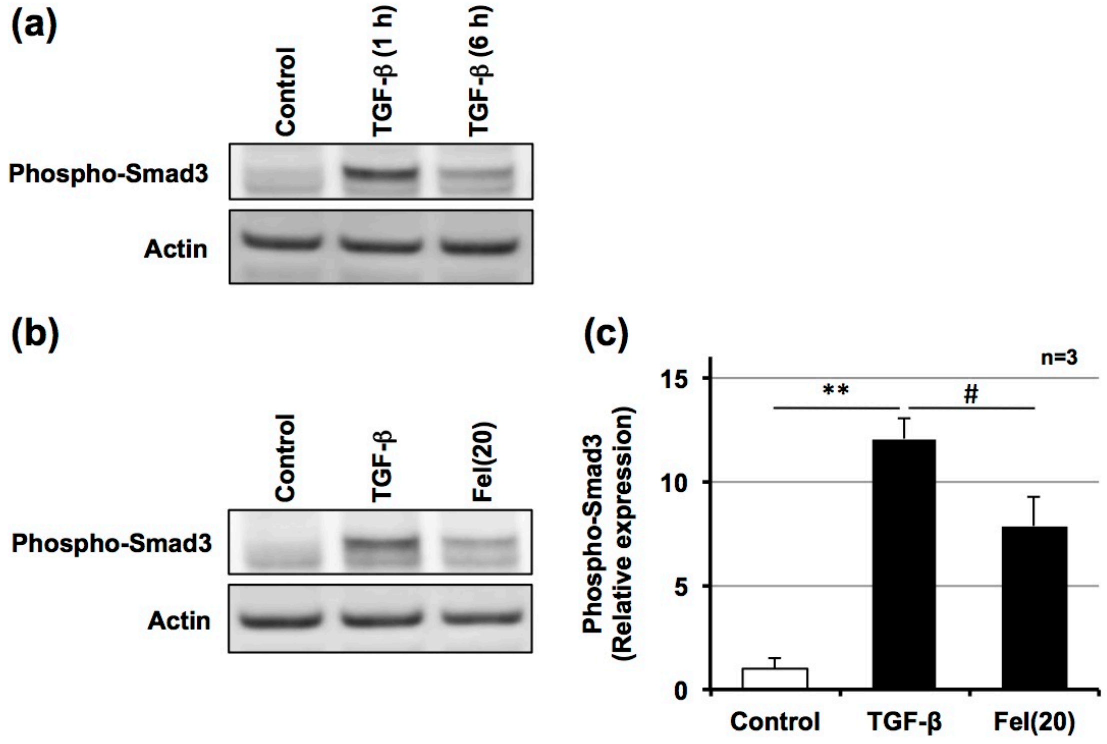

Supplementary Fig. S2. Effect of felodipine on TGF-β1 induced the expression of phospho-Smad3 *in vitro*.

LL29 cells were incubated with TGF-β1 (5 ng/ml) for indicated periods (a) or 1 h (b) in the absence (a) or presence (b) of felodipine (Fel, 20 μM). Whole-cell extracts were analysed by immunoblotting with an antibody against phospho-Smad3 or actin (a, b). The phospho-Smad3 band intensity was determined using ImageJ software (c). Values represent mean ± S.E.M. $^{**}P < 0.01$ and $^{\#}P < 0.05$. (* vs Control, # vs TGF-β).

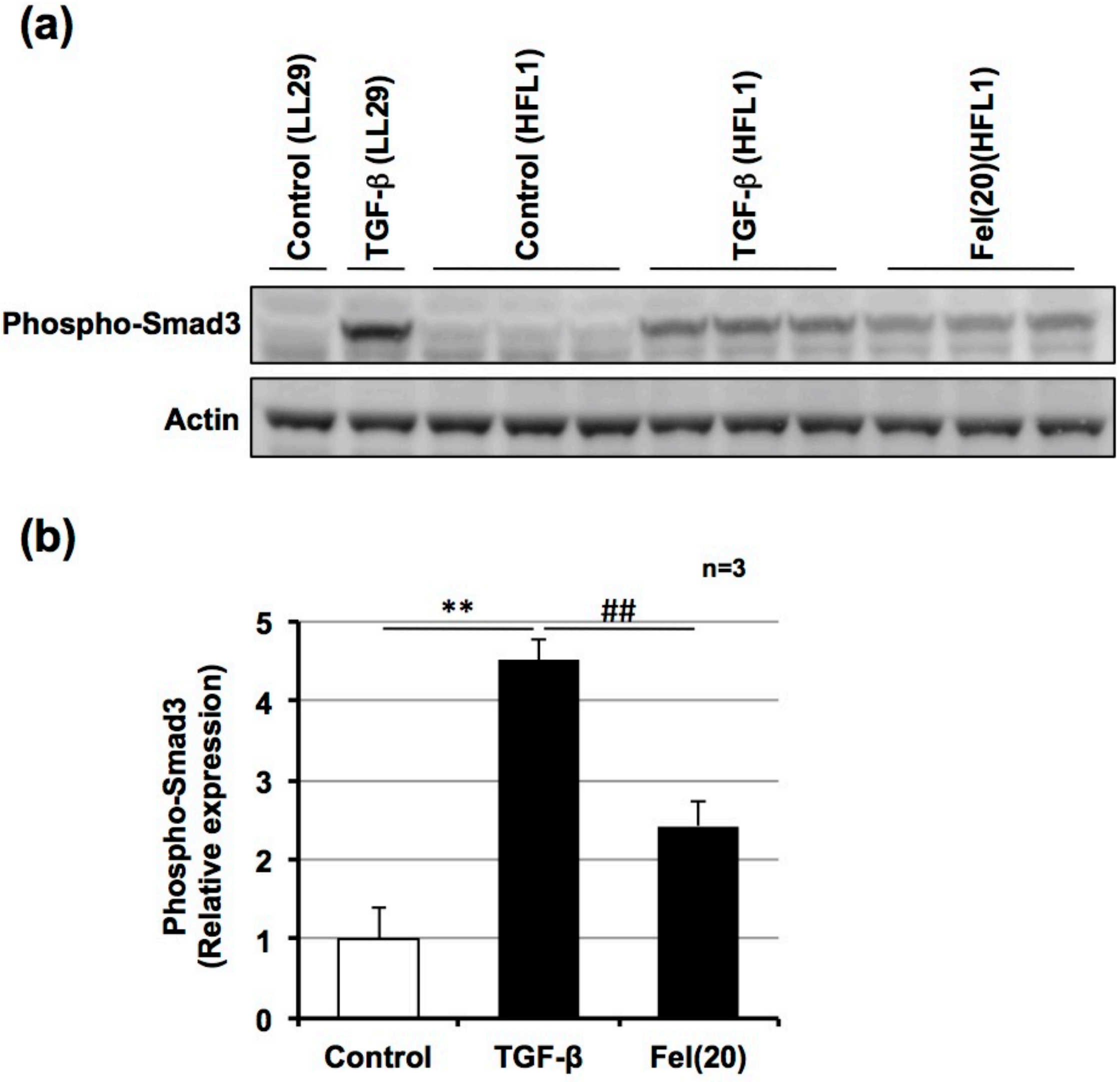

Supplementary Fig. S3. Effect of felodipine on TGF-β1 induced expression of phospho-Smad3 in HFL1 cells.

HFL1 cells or LL29 cells were incubated with TGF-β1 (5 ng/ml) for 1 h in the presence of felodipine (Fel, 20 μM). Whole-cell extracts were analysed by immunoblotting with an antibody against phospho-Smad3 or actin (a). The phospho-Smad3 band intensity was determined using Image J software (b). Values represent mean ± S.E.M. ** or $^{\#\#}P < 0.01$. (* vs Control, # vs TGF).

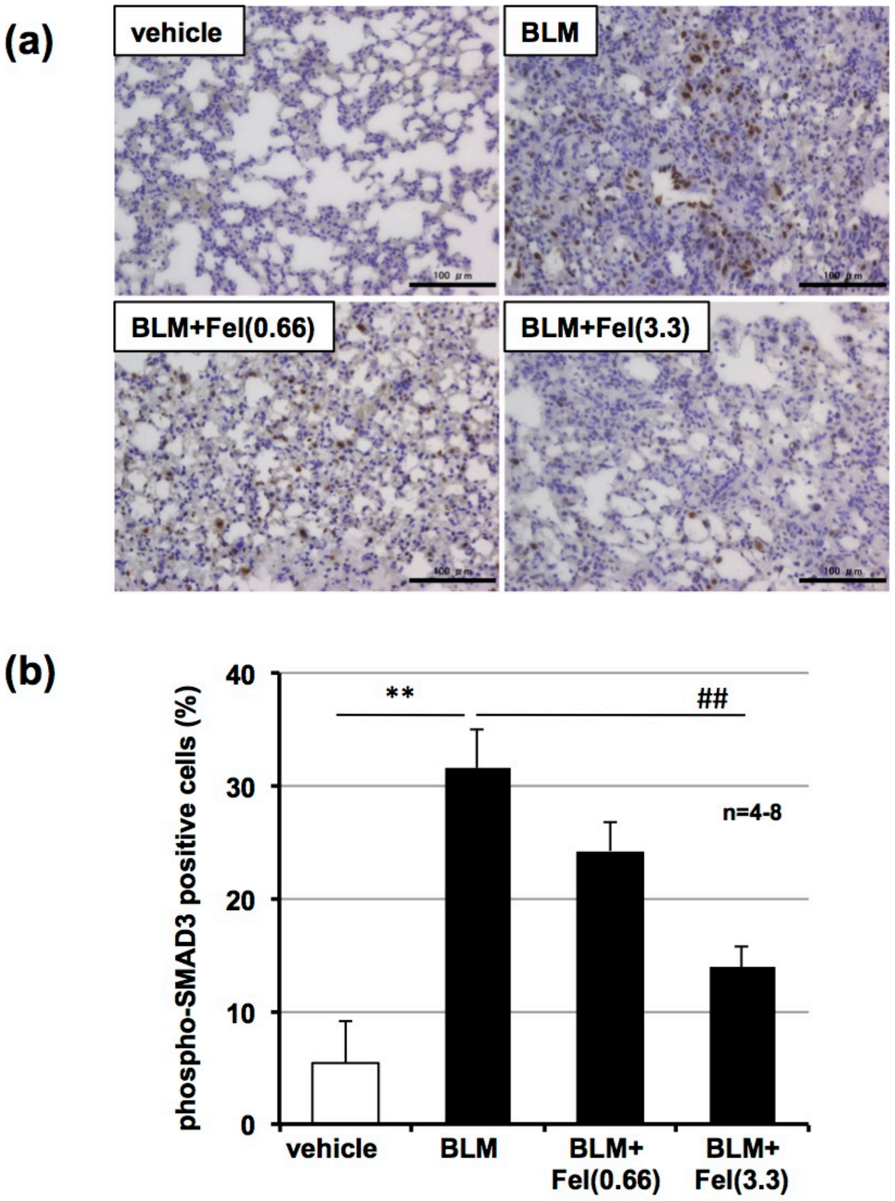

Supplementary Fig. S4. Effect of felodipine on bleomycin induced the expression of phospho-Smad3 *in vivo*.

Mice were intratracheally administered with bleomycin (BLM, 1 mg/kg) or vehicle once only on day 0. Mice were intratracheally administered indicated doses (mg/kg) of felodipine (Fel) once daily for 3 days (from day 10–12). Sections of pulmonary tissue were prepared on day 13 (24 h after final felodipine administration) and subjected to immunohistochemical analysis with an antibody against phospho-Smad3 (a, b). Scale bar = 100 μm. Percentage of area stained with antibody was determined using ImageJ software (b). Values represent mean ± S.E.M. ** or $^{\#\#}P < 0.01$. (* vs vehicle, # vs BLM).

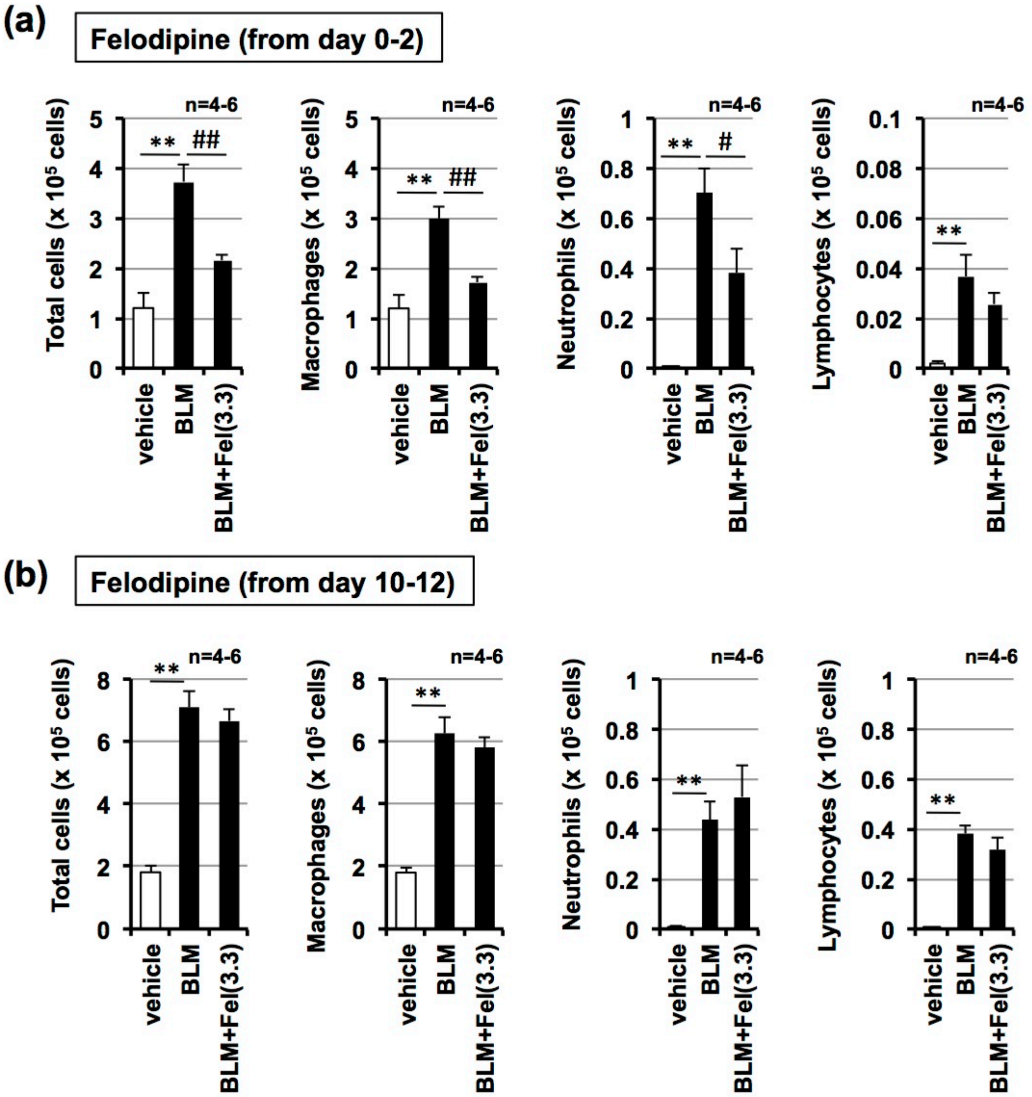

Supplementary Fig. S5. Effect of felodipine on bleomycin-induced pulmonary inflammation.

Preventive study; Mice were intratracheally administered with bleomycin (BLM, 2 mg/kg) or vehicle once only on day 0. Mice were intratracheally administered felodipine (Fel, 3.3 mg/kg) once daily for 3 days (from day 0–2). Bronchoalveolar lavage fluid (BALF) were prepared on day 3 (a).

Therapeutic study; Mice were intratracheally administered with bleomycin (BLM, 1 mg/kg) or vehicle once only on day 0. Mice were intratracheally administered felodipine (Fel, 3.3 mg/kg) once daily for 3 days (from day 10–12). BALF were prepared on day 13 (b).

The numbers of total cells, macrophages, neutrophils and lymphocytes in BALF were determined (a, b). Values are mean ± S.E.M.; ** or $^{\#\#}P < 0.01$ and $^{\#}P < 0.05$. (*, vs vehicle; #, vs BLM).

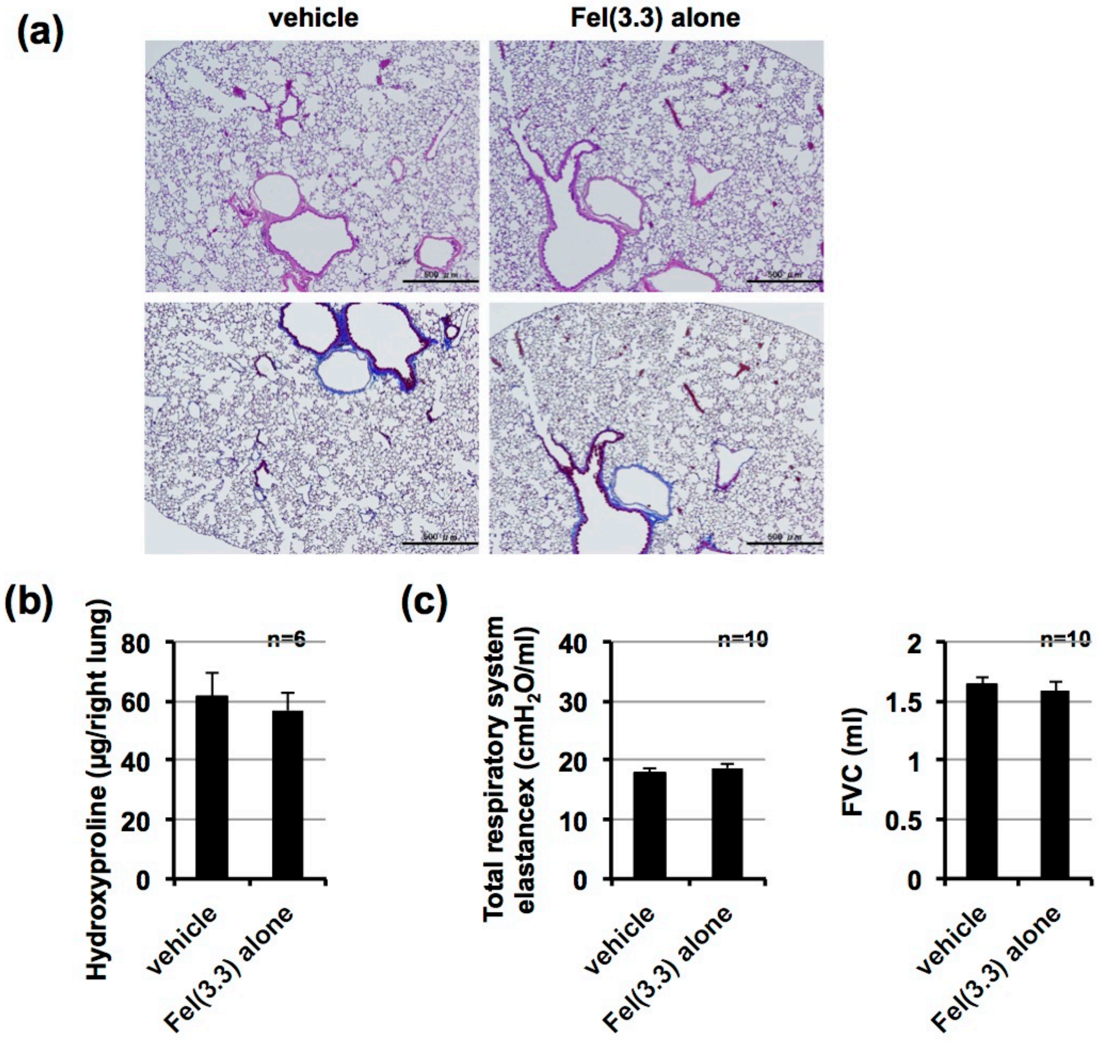

Supplementary Fig. S6. Effect of administering felodipine alone.
Mice were intratracheally administered felodipine (Fel, 3.3 mg/kg) once daily for 14 days (from day 0–13). Sections of pulmonary tissue were prepared on day 14 (24 h after final felodipine administration) and subjected to histopathological examination including H&E staining (upper images) and Masson's trichrome staining (lower images). Scale bar = 500 µm (a). Pulmonary hydroxyproline level was determined on day 14 (b). Total respiratory system elastance and FVC were measured on day 14 (c). Values represent mean ± S.E.M.

Fig.1d

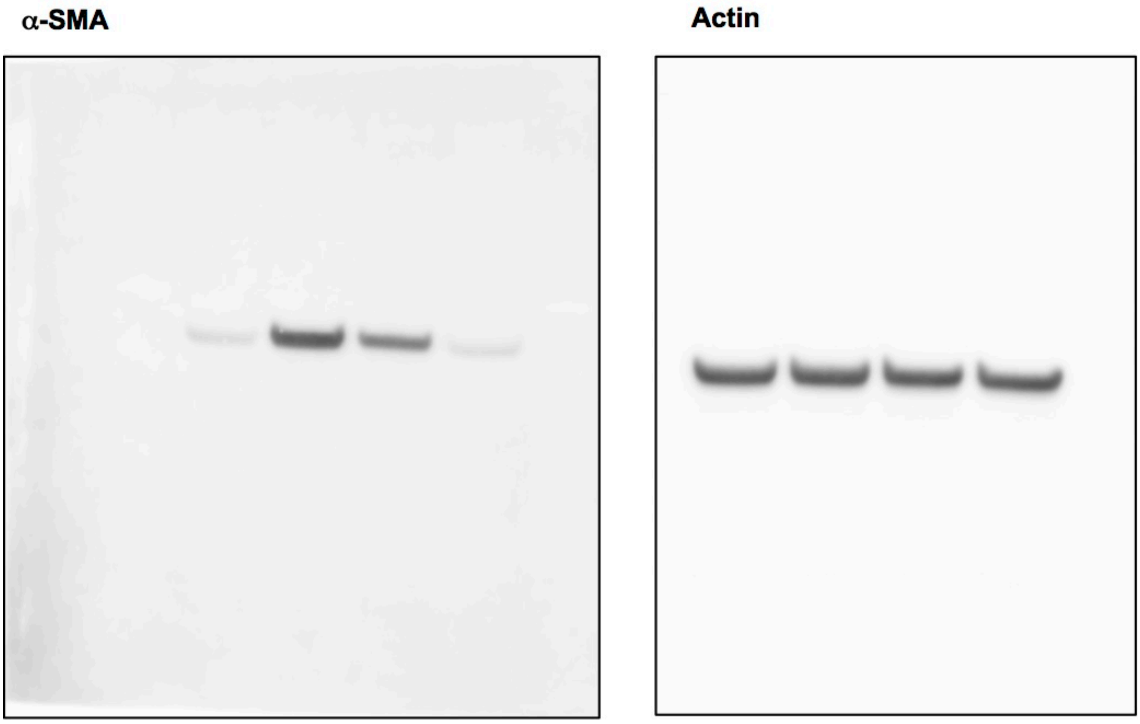

Supplementary Fig. S7. Full gel scans for Fig. 1d.

Supplementary Fig.S1

α-SMA **Actin**

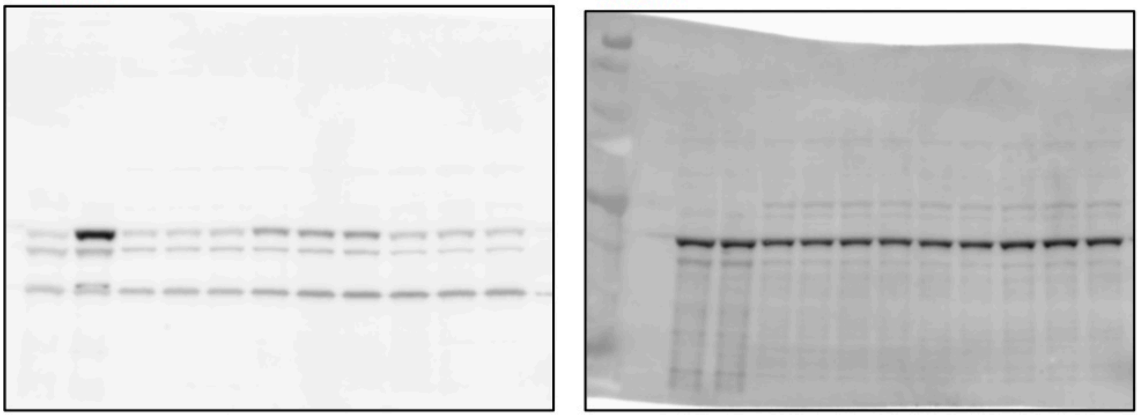

Supplementary Fig.S3

Phospho-Smad3 **Actin**

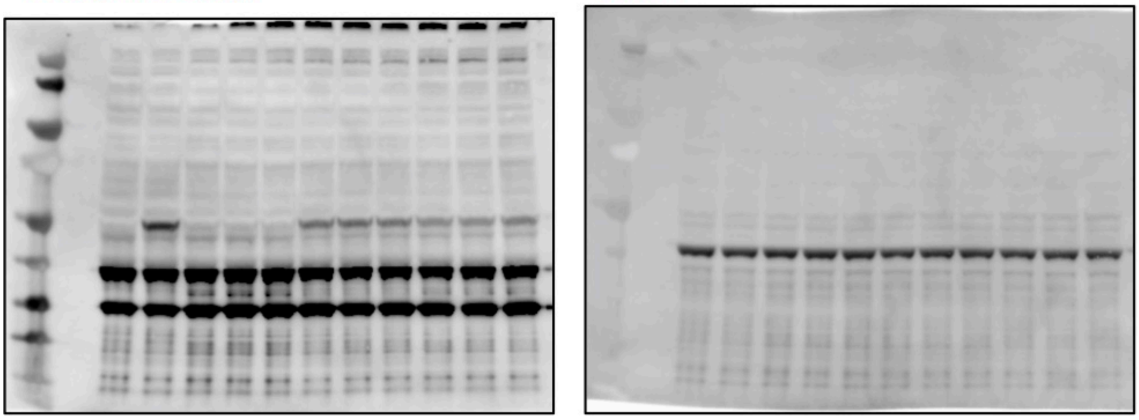

Supplementary Fig. S8. Full gel scans for Supplementary Figs. S1 and S3.

Supplementary Fig.S2a

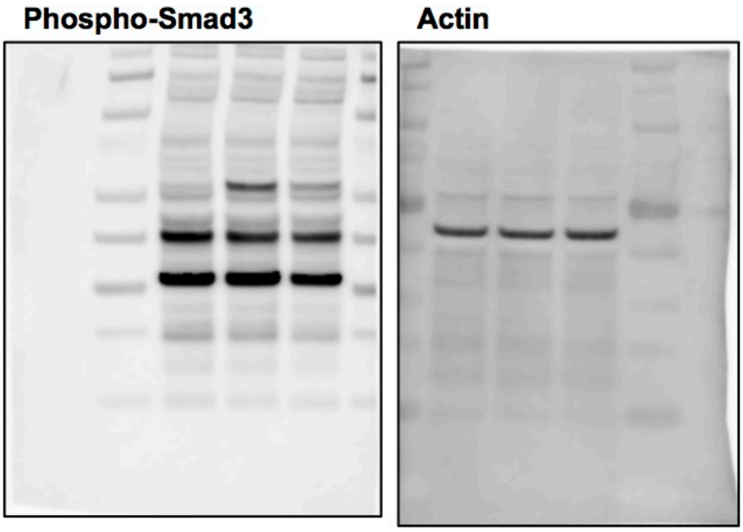

Supplementary Fig.S2b

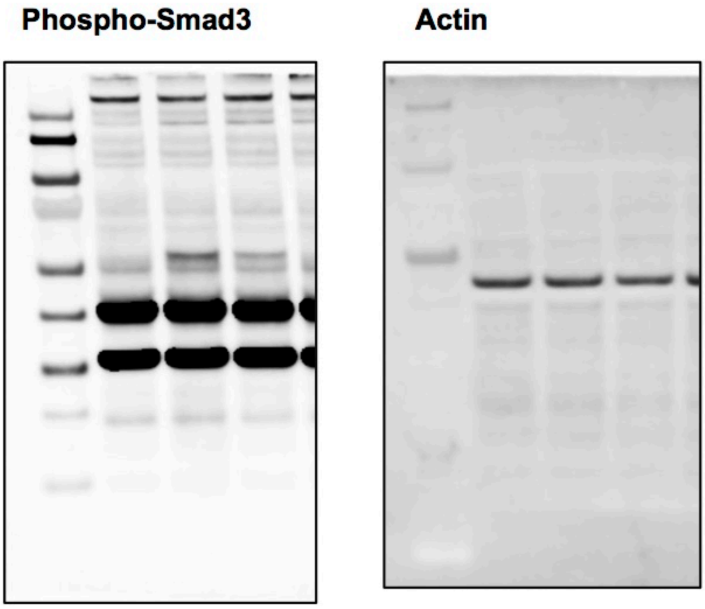

Supplementary Fig. S9. Full gel scans for Supplementary Fig. S2.
